# Regeneration of adhesive tail pad scales in the New Zealand gecko (*Hoplodactylus maculatus*)(Reptilia; Squamata; Lacertilia) can serve as an experimental model to analyze setal formation in lizards generally

**DOI:** 10.24272/j.issn.2095-8137.2017.046

**Published:** 2017-07-18

**Authors:** Lorenzo Alibardi, Victor Benno Meyer-Rochow

**Affiliations:** ^1^Comparative Histolab and Department of Bigea, Dipartimento di Biologia, University of Bologna, Bologna 40126, Italy; ^2^Research Institute of Luminous Organisms, Tokyo 100-1623, Japan; ^3^Department of Genetics and Physiology, Oulu University, Oulu, FIN 90140, Finland

**Keywords:** Gecko lizard, Regeneration, Epidermis, Tail pad scales, Adhesion, Prehensile function, Ultrastructure

## Abstract

During the regeneration of the tail in the arboreal New Zealand gecko (*Hoplodactylus maculatus*) a new set of tail scales, modified into pads bearing setae 5-20 μm long, is also regenerated. Stages of the formation of these specialized scales from epidermal pegs that invaginate the dermis of the regenerating tail are described on the basis of light and electron microscopic images. Within the pegs a differentiating clear layer interfaces with the spinulae and setae of the Oberhäutchen according to a process similar to that described for the digital pads. A layer of clear cytoplasm surrounds the growing tiny setae and eventually cornifies around them and their spatular ends, later leaving the new setae freestanding on the epidermal surface. The fresh adhesive pads help the gecko to maintain the prehensile function of its regenerated tail as together with the axial skeleton (made of a cylinder of elastic cartilage) the pads allow the regenerated tail to curl around twigs and small branches just like the original tail. The regeneration of caudal adhesive pads represents an ideal system to study the cellular processes that determine setal formation under normal or experimental manipulation as the progressive phases of the formation of the setae can be sequentially analyzed.

## INTRODUCTION

The regeneration of the tail in lizards involves the regrowth of a variety of tissues, of which the skin with its regenerated (neogenic) scales is one type (Alibardi & Meyer-Rochow, 1988, 1989; Bellairs & Bryant, 1985; Maderson et al., 1978). The new scales are formed through an initial morphogenetic process that is different from the process of scale formation during development, since the initial regenerating (wound) epidermis undergoes invagination and in the dermis forms pegs that ultimately give rise to the new scales (Alibardi, 1995; Bryant & Bellairs, 1967; Liu & Maneely, 1969; Wu et al., 2014). The regenerated scales in numerous lizard species appear to be of similar shapes and patterns of arrangement, and they are usually smaller than the original scales, allowing the regenerated tail to be distinguished from the original tail.

**Figure 1:**
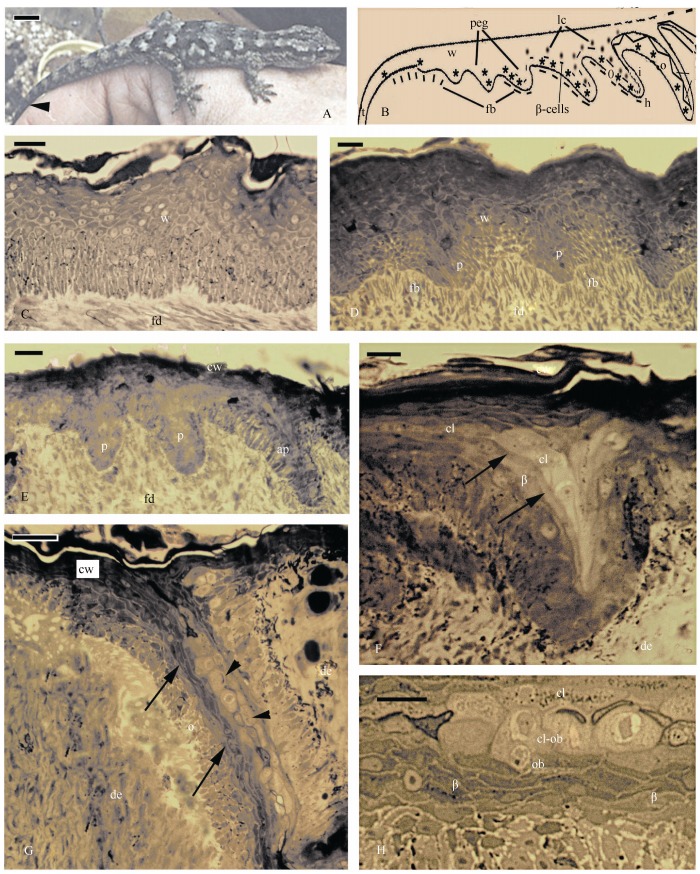
Images and histological aspect of the regenerating tail skin of *Hoplodactylus maculatus*

In some geckos, specialized scales are also regenerated like, for instance, the large, dorsal plate-like scales in *Teratoscincus* (Werner, 1967), or the caudal adhesive pads in *Lygodactylus* (Maderson, 1971) and various Carphodactyline geckos (Bauer, 1998). In the latter scales, especially present on the ventral side of the tail, the external layer exhibits micro-ornamentation, brought about by the so-called Oberhäutchen, and features long bristles like those present in the digital pads that allow caudal adhesion and permit arboreal climbing in these geckos (Hiller, 1972; Maderson, 1966). Studies on scale regeneration have indicated six main stages in the histology of the epidermis: stage 1 indicative of the resting phase, and stages 2-6 covering the renewal period. During scale regeneration these stages are repeated and the new scales pass through similar differentiating stages to those normally occurring during the shedding cycle, forming an external corneous wound epidermis (lacunar cells), followed by a clear layer, the Oberhäutchen, and then a beta-, meso- and alpha-layer (Figure 1A, B).

Histological studies on the regeneration of caudal pads in *Lygodactylus picturensis* have shown the formation of setae in the lamellae of these modified scales (Maderson, 1971), but this study did not provide cytological details on the process of setal formation, in particular on the interaction between clear and Oberhäutchen cells in forming the setae as described for the digital pads (Alibardi, 1999, 2009; Alibardi et al., 2011; Hiller, 1972). However, to possess this information is important, since it is believed that the knowledge of the proteins forming the cytoskeleton in clear cells is essential to understand how these same cells can mould the spinulae and setae of the Oberhäutchen cells, forming the species-specific micro-ornamentation and setal branching patterns in geckos and other lizards that are equipped with such adhesive pads.

In order to verify some cytological details of setal regeneration in the caudal pads, we used samples of the New Zealand gecko (*Hoplodactylus maculates*), collected by us in the field. The geckos possessed regenerated scales at the tip of the new tail (Bauer, 1998), suggesting that the regenerated tail can also make use of these pads for adhesion and movement among a tree's branches. In order to fulfil its role in climbing, the tail must be capable to curl nearly as well as the original tail, and indeed this does occur since the regenerated axial skeleton of the regenerated tail is composed of a tube of elastic cartilage (Alibardi & Meyer-Rochow, 1989). In the present study, we focus on the regenerating tail skin, which allows us to find some stages of setal regeneration and to conclude that this is a promising experimental system to analyze details of setal formation in lizards generally.

## MATERIAL AND METHODS

A total of six individuals of the New Zealand gecko, *H. Maculatus*, with regenerating tails of 3-4 mm (*n*=4) and about 10 mm (*n*=2) were used in the present study (Figure 1A). Details of animal collection and fixation were previously provided (Alibardi & Meyer-Rochow, 1988, 1989). Autotomy, a natural mechanism of tail release following grabbing of the tail, was induced, and the regenerating tail was collected at 40 days (*n*=4) and 60 days (*n*=2) of regeneration when scalation was visible. Permission to carry out the research and approval of the experimental protocol was obtained from the institutional Ethics Committee on Animal Care and Welfare of the University of Waikato (Hamilton; New Zealand).

In these samples, the regenerating tail showed stages of epidermal differentiation spanning from the beginning of scale formation to completely formed scales over most of the surface of the regenerated scales. Among the normal caudal scales a few differentiated pad lamellae were also present, in particular noticeable at 60 days of regeneration. Briefly, for plastic embedding, small pieces of tail tissues from four individuals were initially fixed in 2.5% glutaraldehyde in cacodylate buffer for about eight hours, osmicated, dehydrated and included in Epon. For wax embedding, the tissues from two individuals were fixed in 10% buffered formaldehyde for about 12 hours, dehydrated, cleared with xylene, and embedded in wax.

Sections 2-3 μm thick form plastic-embedded tissues were collected using an ultramicrotome (Nova, LKB, Bromma, Sweden), dried on a glass slide and stained with 1% toluidine blue solution. Interesting levels of the tissues, showing most likely the presence of differentiating Oberhäutchen and beta-cells, were sectioned at 40-80 nm thickness with an ultramicrotome and were collected on 200 mesh copper grids, stained for 30 minutes at room temperature with 2% uranyl acetate, washed and stained for 6 minutes in lead citrate according to standard procedures. The sections were observed under a Zeiss C10 transmission electron microscope at a high tension of 60 kV. Images were collected on a digital camera and imported into a computer, allowing representative section images to be used in composing figures.

Wax sections of 6-8 μm thickness, obtained with the help of a rotary microtome (Reichert, Germany), were dried on glass slides for some hours and stained with 1% toluidine blue for a minute. Pictures were taken under a light microscope equipped with a digital camera, imported into a computer and selected to compose the figures.

## RESULTS

### Histology of the regeneration of pad scales

The four available specimens with poorly scaled regenerating tails of 3-4 mm in length, showed some stages (2-4) of the scale regeneration sequence (indicated in Figure 1B). Initially the thick wound epidermis toward the tip of the tail was linear or undulated (Figure 1C), but in more proximal regions epidermal pegs were formed and they became asymmetric (with a longer, distal side) toward the tail stump (Figure 1D, E). Inside these pegs, underneath the dark corneous layer of the wound epidermis, the differentiation of clear and darker beta-cells started in the middle of the elongated peg, indicative of stages 3-4 of the shedding cycle (Figure 1F). The Oberhäutchen layer was sandwiched between the clear and darker beta-cells, representing the first line of cells contacting the hyperthrophic and pale clear cells (Figure 1G, H).

This early stage of differentiation (stage 3-4: Maderson, 1966, 1970) did not allow us to detect the tiny spinulae and setae originating from Oberhäutchen cells, but small granulations and seemingly irregular filaments were seen inside the pale cells, giving a granular appearance to their cytoplasm (Figure 1G). The external, corneous wound epidermis covering the entire regenerated epidermis in more proximal regions was detached from the pegs, being evidence of the beginning of the shedding process at stage 5 of the shedding cycle (Figure 1F, G). No further stage of setal formation was apparent, but this same material, analyzed under the electron microscope, revealed some important details (see further below).

**Figure 2:**
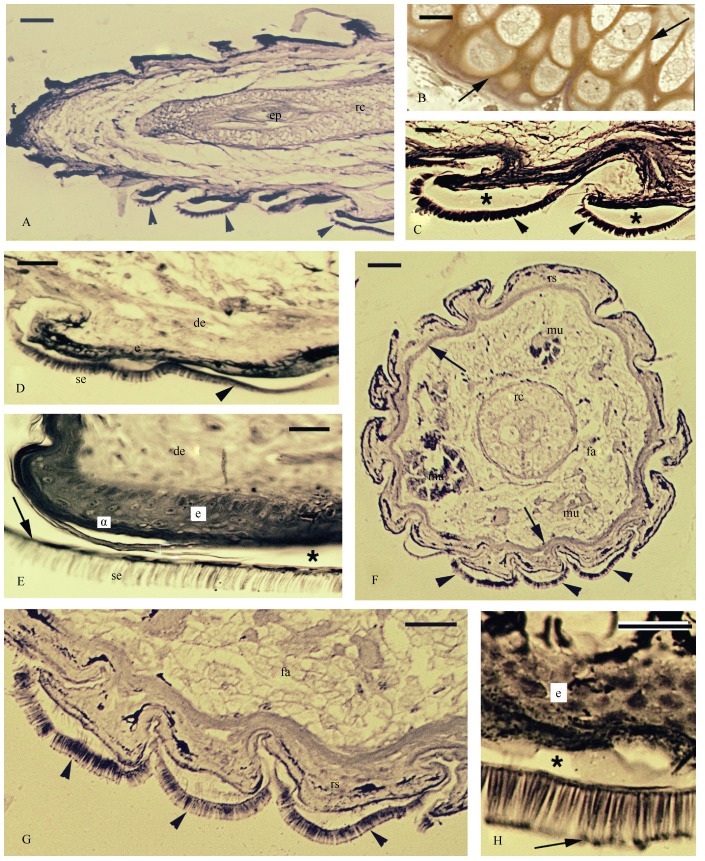
Histological aspect of regenerating tail with formed tail pad lamellae

An examination of the two advanced regenerated tails, about 10 mm long (60 days regeneration), one sectioned sagittally and the other transversely, showed that they were completely scaled and contained an axial tube of elastic cartilage surrounding the ependymal tube (Figure 2A, B). Toward the tip of the tail on its ventral side (this was determined by an examination of the regenerating cartilage in relation to the vertebrae of the tail stump), the presence of lamellar pads bearing bristles among the normal scales was noted, whose outer (dorsal) surface appeared decorated with indentations not seen in the remaining scales (Figure 2A, C, D). A closer look at higher magnification showed that all these pads appeared at the post-shedding, resting stage or stage 2 of the shedding cycle with completely mature setae and beta-layer, while the alpha layer was still uncompleted (Figure 2E).

The localization of the lamellar pads was better appreciated in cross sections, which confirmed that these modified and indented scales were restricted to the ventral side, forming three or four rows of scales (Figure 2A, F, G). The maximal length of the setae was approx. 20 μm. The enlargements of their terminal tips were probably related to the apical branching into thinner setae and the development of adhesive spatulae (Figure 2H).

In summary, while at the early stages of scale regeneration (40 days regeneration) only the beginning of setal formation was detected, at the later stage (60 days regeneration) mature setae were present.

### Ultrastructure of setal formation

**Figure 3:**
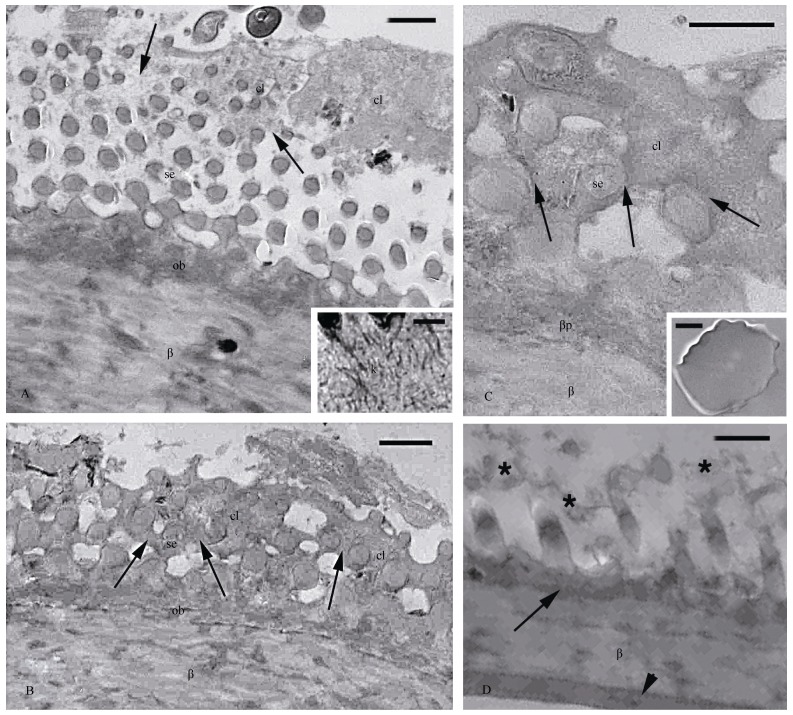
Electron microscopy images (TEM) aspect of formed setae in regenerated scales

Although the progressive formation of setae in its entirety was not seen in the available material, careful examination of the epidermis at stages 3-4 of the shedding cycle detected in the elongated pegs the pale cytoplasm of clear cells, which surrounded the numerous, tiny spinulae and the setae formed from the Oberhäutchen layer. The more proximal, normal scales showed a completely formed beta-layer (stage 5, close to shedding) merged to the Oberhäutchen from which protruding spinulae of 0.2 by 1.0 μm were present (data not shown). Only short setae were seen in the sparse pads sectioned in the available material and they were slightly thicker and clearly longer than the spinulae, e.g., 0.3-0.6 μm versus 4-5 μm or more (Figure 3A). Among the spinulae or the setae, the pale cytoplasm of the clear cells with a loose meshwork of cytoskeletal filaments mainly composed of keratin (diameter=10 nm; inset of Figure 3A) was apparent. In other regions of the tail pad lamella, the cytoplasm of the clear cells became dense and fibrous, especially around each seta (Figure 3B, C). In the regenerated and more proximal scales, after shedding of the corneous wound epidermis (Figure 1F, G), the cytoplasm of the clear cells was either totally or partially degenerated among the spinulae or the longer setae, which were therefore free-standing on the skin surface (Figure 3C inset, D). As in other geckos (Bauer,1998; Hiller, 1972; Maderson, 1971), the beta-layer sustaining the mature setae also in *H. maculatus* appeared subdivided into two darker strata and one pale stratum sandwiched between them (Figure 3D).

In summary, in the regenerating scales at stages 3-4 the formation of setae in the caudal pads resembled the typical process that occurs during the formation of digital scales with mature setae at the late stages resting upon a merged Oberhäutchen and beta-layer.

## DISCUSSION

Although the available stages of regeneration in the adhesive caudal pads were incomplete to describe the entire process of setal differentiation, the combination of light and electron microscopic observations revealed the novel finding that the formation of setae in the caudal pads largely resembled that of the digital pads earlier described by Hiller (1972), Alibardi (1999, 2009) and Alibardi et al. (2011). Thin setae, 0.3-0.6 μm in diameter and 5-20 μm in length, with terminal branches into thin spatulae, were present, as also previously reported (but based solely on scanning electron microscopy (SEM) images (Bauer, 1998) in some ventral scales at the tip of the tail of *H. maculatus*. However, mainly tiny setae were apparent and only in a few cases the thicker basal parts of mature setae 0.9-1.2 μm thick or their characteristic small spatula ends were detected. Whether functionally and chemically the regenerated setae are identical to the original setae of normal scales is something that we cannot say without further studies.

**Figure 4:**
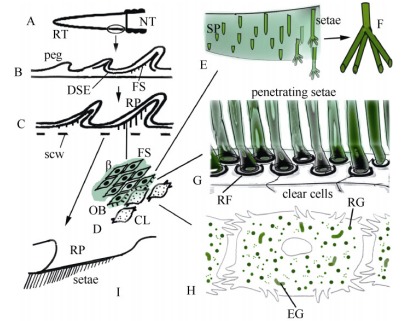
Schematic drawing illustrating the formation of setae in the caudal pads of the gecko

As for the digital setae (Alibardi, 1999; Alibardi et al., 2011; Hiller, 1972), also those of the regenerating tail pads utilize the guidance of the cytoplasm of the clear cells (Figure 4) to extensively branch into very tiny setal ends (spatulae) that likely gift these caudal pads with adhesion properties with the same efficiency as the digital pads. The large pale cells occupying the position of the clear cells (indicated as cl-ob in Figure 1G), resemble the beta-glandular cells previously described in geckos (beta-glands, cf., Maderson, 1970). However the tiny granulations present in these cells (Figure 1H) may actually represent very thin intra-cytoplasmic branching of Oberhäutchen setae within the cytoplasm of the clear cells, but the lack of the sequence of differentiating cells precluded a clarification of this important issue (Figure 4). The presence of very large and roundish pale cells, referred to as clear cells and containing granulations, was also described during setal formation in the gecko *Tarentula mauritanica* (Hiller, 1972). The nature of this intimate, almost symbiotic penetration of numerous setae into the cytoplasm of the clear cells could be confirmed by our ultrastructural observations (Figure 3A), and is schematically represented in Figure 4.

The ultrastructural study has also shown that in tail pads the fibrous cytoskeletal material formed in the clear cells surrounds the setae during their formation and eventually becomes cornified. In normal caudal scales, the maturation of the corneous layer and its shed occur through the detachment of the corneous clear layer from the Oberhäutchen, but in the case of the setae formed in the caudal pads it appears that the corneous and dead clear layer degenerates between the numerous setae after the other corneous layers of the wound epidermis have been shed (Figure 3D, Figure 4A, C). It remains unclear, however, whether shedding in this specialized tail scale type occurs by the detachment of the clear Oberhäutchen layer as in the digital scales, or is instead due to the degeneration of the cytoplasm of the clear cells. Whatever the scenario, the disappearance of the clear layer allows the setae to become free and exposed to the substrate effectuating adhesion.

## CONCLUSION

The present study demonstrates that the process of setal formation in regenerating caudal pads in geckos can serve as a useful experimental system to analyze details of setal formation generally since the regeneration of scales and pads follows a proximal-distal direction of differentiation, along which all the stages of the typical renewal phase of the epidermal shedding cycle are present (Alibardi, 1995; Maderson, 1971). However, the interest in adhesive pads goes beyond that, because there are potential applications for using information gained from such scales and their setal properties in biomimetics to produce the next generation of dry adhesives. The study of regenerating adhesive pads during tail regeneration in geckos (at 40 and 60 days in *H. maculatus*) is ethically and ecologically acceptable, as it does not require any sacrifice of animals (except for the tail, which, however, will have regenerated and regained its full function within a few months after its loss), and at the same time would allow to perfectly stage all the phases of setal formation and enable a detailed investigations of the functional properties of the adhesive pads and their setae.

## ACKNOWLEDGEMENTS

The study was completely self-supported (Comparative Histolab), while L.A.'s trip to New Zealand and stay at V.B.M-R.'s lab at the University of Waikato were largely supported by a New Zealand University Grants Committee Scholarship.
